# Enhancing mesenchymal stem cell survival and homing capability to improve cell engraftment efficacy for liver diseases

**DOI:** 10.1186/s13287-023-03476-4

**Published:** 2023-09-04

**Authors:** Shaoxiong Yu, Saihua Yu, Haiyan Liu, Naishun Liao, Xiaolong Liu

**Affiliations:** 1 The United Innovation of Mengchao Hepatobiliary Technology Key Laboratory of Fujian Province, Mengchao Hepatobiliary Hospital of Fujian Medical University, Fuzhou, 350025 People’s Republic of China; 2 Mengchao Med-X Center, Fuzhou University, Fuzhou, 350116 People’s Republic of China; 3 The Liver Center of Fujian Province, Fujian Medical University, Fuzhou, 350025 People’s Republic of China

**Keywords:** Mesenchymal stem cells, Cell survival, MSC homing, Engraftment efficiency, Liver diseases

## Abstract

Although mesenchymal stem cell (MSC) transplantation provides an alternative strategy for end-stage liver disease (ESLD), further widespread application of MSC therapy is limited owing to low cell engraftment efficiency. Improving cell engraftment efficiency plays a critical role in enhancing MSC therapy for liver diseases. In this review, we summarize the current status and challenges of MSC transplantation for ESLD. We also outline the complicated cell-homing process and highlight how low cell engraftment efficiency is closely related to huge differences in extracellular conditions involved in MSC homing journeys ranging from constant, controlled conditions in vitro to variable and challenging conditions in vivo. Improving cell survival and homing capabilities enhances MSC engraftment efficacy. Therefore, we summarize the current strategies, including hypoxic priming, drug pretreatment, gene modification, and cytokine pretreatment, as well as splenectomy and local irradiation, used to improve MSC survival and homing capability, and enhance cell engraftment and therapeutic efficiency of MSC therapy. We hope that this review will provide new insights into enhancing the efficiency of MSC engraftment in liver diseases.

## Introduction

End-stage liver diseases (ESLD), including decompensated liver cirrhosis, liver failure, and hepatocellular carcinoma, have high mortality rates, and their prevalence has increased in recent years [1]. ESLD is characterized by severely abnormal liver functions including hepatic decompensation, portal hypertension, coagulation dysfunction, jaundice, hepatorenal syndrome, hepatic encephalopathy, and ascites. Although liver transplantation can effectively treat these diseases [2], most patients die waiting for transplant surgery because of a shortage of donor organs. Mesenchymal stem cells (MSCs) are adult multipotent cells with self-renewal, multi-directional differentiation, immunoregulator, and paracrine functions [3]. Recent findings have demonstrated that MSC transplantation can improve liver function in acute or chronic liver diseases, offering an alternative strategy for patients with ESLD to prolong their life [4–9]. The therapeutic functions of MSC transplantation are attributed to the following aspects. First, MSCs serve as substitutes for hepatocytes via transdifferentiation or cell fusion for liver tissue repair and regeneration. Second, MSCs exhibit paracrine functions by releasing growth factors and cytokines that inhibit hepatocyte apoptosis and stimulate liver regeneration. Third, MSCs possess immunomodulatory properties related to adaptive and innate immune responses [10].

According to the potential therapeutic mechanisms of MSC therapy, the paracrine or immunoregulatory actions of MSCs depend on their survival rate in vivo, and the hepatic differentiation or fusion function of MSCs depends on the number of viable MSCs that reached the injured liver tissues. Thus, MSC engraftment efficiency is closely related to cell survival or viability and sufficient delivery of cells to the liver. Actually, Kuo et al. found that survival of MSCs in liver tissues was less than 5% 4 weeks after transplantation [11]. Our previous work showed that a large number of MSCs die within 1 day after transplantation in fibrotic liver of mice, and the surviving MSCs almost completely disappeared 11 days after transplantation [12], indicating an extremely low MSC survival rate in vivo, leading to insufficient cell engraftment efficiency for liver diseases. Therefore, cell attrition has become a major bottleneck in MSC therapy for liver diseases. Improving cell survival and MSC homing capability to enhance cell engraftment efficiency is needed to maximize the therapeutic potential of MSC transplantation in liver diseases.

**Fig. 1:**
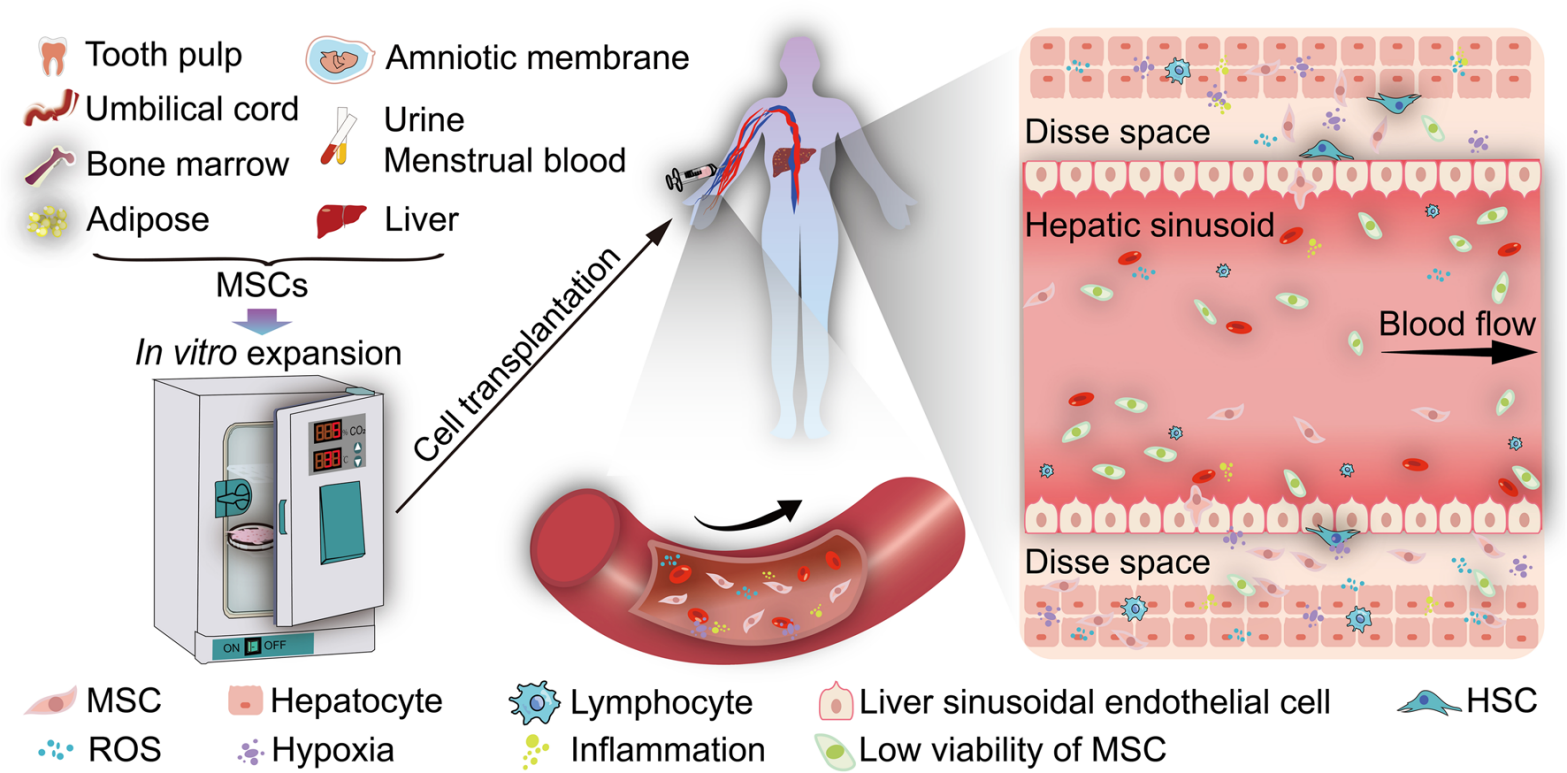
The dramatic difference between in vitro and in vivo conditions in MSC transplantation for liver diseases. The MSC engraftment process is tortuous, and the transplanted MSCs would encounter dramatic changes ranging from in vitro comfortable growing conditions to in vivo inclement environment (such as hypoxia, oxidative stress, and inflammation), leading to the low cell survival of MSC therapy for liver diseases. Figure designed by Adobe Illustrator CC 2018

**Fig. 2:**
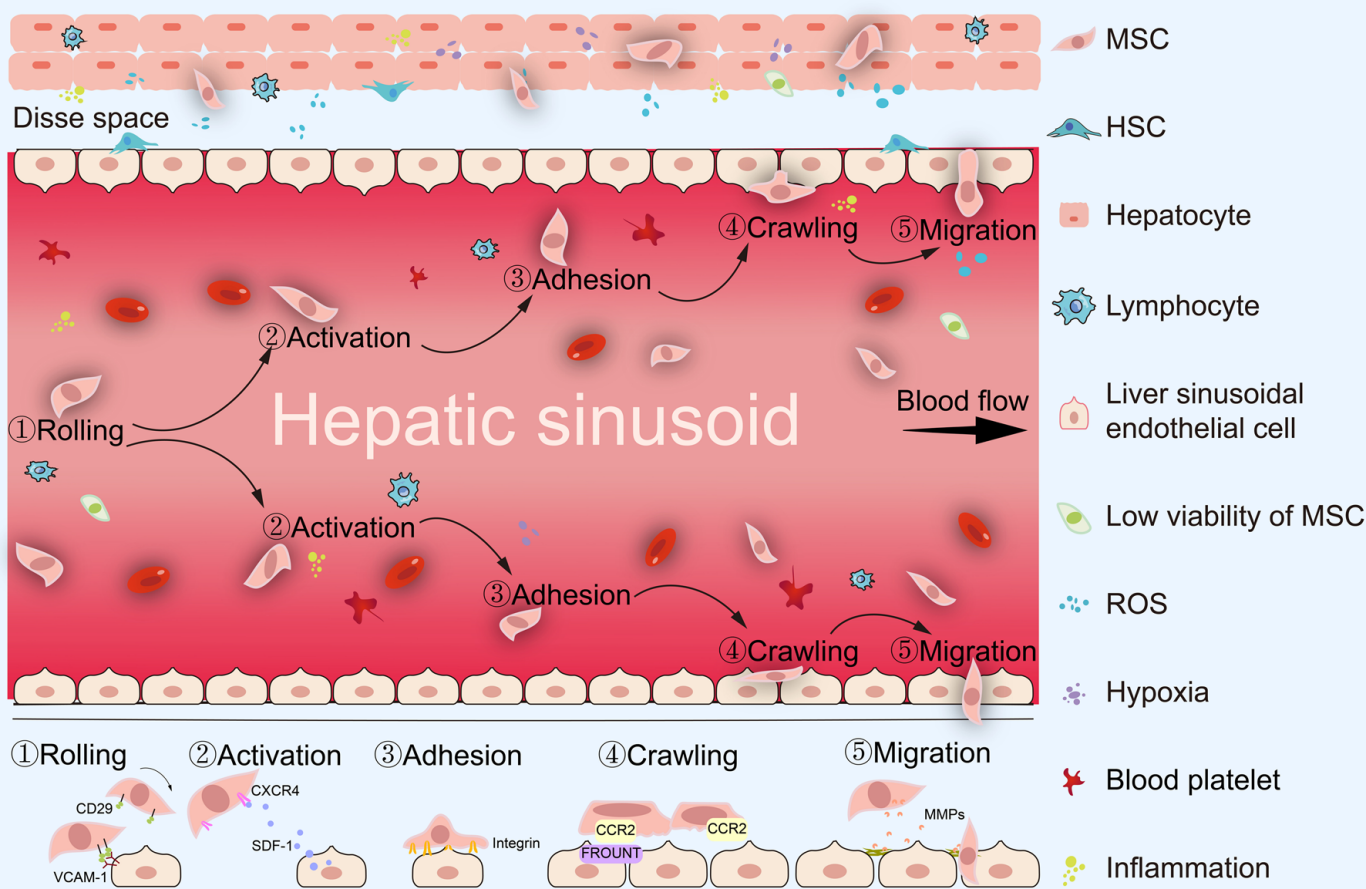
The systemic homing process during MSC therapy. Systemic administration of MSCs must undergo a multistep process including rolling, activation, and adhesion, as well as crawling and migration. Figure designed by Adobe Illustrator CC 2018

Various biological, biochemical, and biophysical factors tightly influence MSC survival and homing capabilities through reciprocal interactions between cells, the extracellular matrix, and bioactive factors both in vitro and in vivo [13]. Dramatically different conditions between in vitro and in vivo severely affect MSC survival or viability after transplantation. During in vitro expansion of MSCs, the conditions are optimally controlled including oxygen partial pressure, whereas MSCs encounter a variety of conditions in vivo, including hypoxia and oxidative stress, that affect their ability to home and effectively repopulated liver tissue during transplantation (Fig. 1). Each part of the homing process comprising rolling, activation, adhesion, crawling, and migration (Fig. 2) affects the number of the homing of MSCs to parenchymal liver tissues. Therefore, regulation of biological, biochemical, and/or biophysical factors to reduce cell injuries induced by an unfavorable environment in vivo can improve MSC survival, increase MSC homing capability, and enhance MSC engraftment efficiency. In this review, we discuss the current status of MSC therapy, the detailed cell-homing process, strategies to improve MSC survival, and homing capability to enhance MSC engraftment efficiency in liver diseases.

## Current status and challenges of MSC transplantation for ESLD

MSCs are adult and multipotent stromal cells that can be isolated from the bone marrow, adipose tissues, umbilical cord, dental tissue, synovium, placenta, and dermis [14]. According to the International Society for Cell and Gene Therapy, MSCs are defined by the following criteria: (1) the cells are adherent under standard culture conditions and grow intrinsically during in vitro expansion or culture; (2) the cell surface makers are positive for CD73, CD90, and CD105, but negative for CD14, CD34, CD45, and HLA-DR; and (3) the cells can differentiate into adipocytes, osteoblasts, or chondrocytes in vitro. In addition to the above properties, MSCs also have immune evasion ability due to low MHC-I antigen expression and lack of MHC-II antigen expression [15], which is a congenital factor for allogeneic or autogenous MSC transplantation [16]. MSC transplantation has been widely used in preclinical studies, for treating ESLD, including liver failure and cirrhosis, indicating its potential for ESLD in humans. A growing number of clinical trials have confirmed the therapeutic potential of MSC transplantation for ESLD, particularly for decompensated liver cirrhosis (DLC) and acute-on-chronic liver failure (ACLF) (Table 1). Li et al*.* found that the overall survival at 3-year (83.3% vs 61.8%) and 5-year (63.9% vs 43.6%) during the 13- to 75-month follow-up was significantly improved by human umbilical cord blood-derived MSC therapy for DLC patients (*n* = 36) [17]. In a randomized controlled clinical trial, MSC transplantation improved overall survival and liver function biomarkers (albumin, prothrombin activity, cholinesterase, and total bilirubin during 48 weeks of follow-up) during a 13–75-month follow-up in patients with DLC (*n* = 108) [18]. No significant side effects or cell-related complications have been observed after MSC therapy in patients with DLC. A randomized controlled trial conducted by Lin et al*.* found that MSC therapy could increase 24-week survival rates (73.2% vs 55.6%) by improving liver function (total bilirubin and MELD scores) and decreasing the incidence of severe infections (16.1% vs 33.3%) in ACLF patients (*n* = 56) [19]. However, Mohamadnejad et al*.* found that in a randomized controlled trial (*n* = 27, 12 months of follow-up) involving patients with cirrhosis, MSC transplantation did not improve child scores, MELD scores, serum albumin, INR, serum transaminases, or liver volumes [20]. Given the fact that the background and guidelines for liver diseases worldwide are not fully uniform, some large, multicenter clinical trials with long-term follow-up in MSC therapy for subcategories of liver diseases should be conducted to further confirm the clinical benefit of MSC therapy.

**Table 1:** Clinical trials of MSC transplantation for ESLD

Liver disease	Cell type	N	Injected site	Phase	Cell dose	Follow-up	Out come	References
DLC	UC-MSC	36	PVI	I/II	0.5–1 × 10^6^/kg/3 times, allogeneic	10 years	3–5 years survival rate was significantly improved	[17]
HBV-DLC	UC-MSC	108	PVI	I/II	0.5 × 10^6^/kg/3 times, allogeneic	75 months	ALT, Tbil, CHE, and MELD scores were significantly improved; long-term survival was significantly improved	[18]
HBV-ACLF	BM-MSC	56	PVI	I	1.0–10 × 10^5^/kg/4 times, allogeneic	24 weeks	Tbil and MELD scores were markedly improved; the incidence of severe infection was decreased	[19]
LC	BM-MSC	25	PVI	I	1 × 10^6^/kg/1 time, autologous	6 months	MELD scores and ALB were improved; unmeasured HCV RNA level; hepatitis activity index scores were decreased	[21]
DLC	BM-MSC	15	PVI	I	1.2–2.95 × 10^8^/people/1 time, autologous	1 year	No significant improvement	[20]
LC	BM-MSC	4	PVI	I	31.73 × 10^6^/kg, autologous	1 year	MELD scores were improved; no side-effects	[97]
LC	BM-MSC	12	PVI	I/II	5.20 ± 0.63 × 10^9^/people, autologous	2 years	Child–Pugh scores were significantly improved; α-Fetoprotein and PCNA were significantly elevated	[98]
HCV-LC	BM-MSC	20	ISI	I	1 × 10^7^/people/1 time, autologous	6 months	Tbil, AST, ALT, PT, and INR levels were decreased; ALB and PC were significantly increased	[99]
Alcoholic-LC	BM-MSC	12	IAI	II	5 × 10^7^/people/1 time, autologous	1 year	Child–Pugh scores were improved; TGF-β1, type 1 collagen, and α-SMA were significantly decreased	[100]
PBC	UC-MSC	5	PVI	I	0.5 × 10^6^/kg/3 times, autologous	1 year	Serum alkaline phosphatase and γ-glutamyltransferase levels were increased; no obvious side-effects	[101]
HBV-LC	UC-MSC	30	PVI	I/II	0.5 × 10^6^/kg/3 times, autologous	1 year	The ascites were significantly reduced; ALB was increased; Tbil and MELD scores were decreased	[102]
PBC	UC-MSC	10	PVI	I	3–5 × 10^5^/kg/1 time, allogeneic	1 year	ALT, AST, g-GT, IgM, and CD8^+^ T cells were reduced; CD4^+^ CD25^+^ Foxp3^+^ T cells and IL-10 were increased	[103]
HCV-LC	BM-MSC	15	PVI	II	10^6^/kg/1 time, autologous	6 months	Prothrombin concentration and ALB were increased; Tbil and MELD scores were decreased	[104]
DLC	BM-MSC	8	PVI *n* = 2 PI *n* = 6	I/II	3.0–5.0 × 10^7^/people/1 time, autologous	24 weeks	MELD scores, prothrombin complex, serum creatinine, ALB, and Tbil were decreased	[105]
LC	BM-MSC	10	PVI	I	5.20 ± 0.639 × 10^9^/people/1 time, autologous	4 months	ALB, total protein, Child–Pugh scores, Alpha-fetoprotein, and PCNA were improved	[106]
HCV-HCC	BM-MSC	20	PVI	I/II	1 × 10^6^/kg/1 time, autologous	1 year	ALB, Tbil, INR, PC, and ALT were significantly improved	[107]
DLC	UC-MSC	50	IAI	I	3 × 10^7^/people/1 time, autologous	24 weeks	ALB and pre-ALB were significantly increased; in the first 2–3 weeks, abdominal distension, oliguria, and edema were decreased	[108]
Alcoholic-LC	BM-MSC	37	IAI	II	5 × 10^7^/people/1–2 time, autologous	12 months	The proportion of collagen was decreased; Child–Pugh scores were significantly improved; no side effects	[109]
LC	BM-MSC	1	IAI	I	1.2 × 10^8^/people/2 times, autologous	12 months	Tbil was decreased; ALB was improved; the ascites was reduced	[110]
HBV-DLC	UC-MSC	50	PVI	I/II	4.0–4.5 × 10^8^/people/2 times, allogeneic	52 weeks	Liver function level including ALB, Tbil, and prothrombin were improved during 3–5 weeks; IL-6 and TNF-α were decreased; TGF-β1 and IL-10 were significantly increased	[111]
LC	ADSC	7	IAI	I/II	3.3 × 10^5^/kg/1 time, autologous	24 weeks	ALB and prothrombin activity were improved; no side effects	[112]
LC	ADSC	2	IAI	I	3.3/6.6 × 10^5^/kg/1 time, autologous	1 year	HGF and IL-6 were increased after MSC infusion; ALB were maintained or improved	[113]
LF	BM-MSC	53	IAI	I/II	0.5–1 × 10^6^/kg /1 time, autologous	192 weeks	ALB was increased after 2-week transplantation; the life quality was significantly improved	[114]
HCV-LF	BM-MSC	20	PVI ISI	I	2 × 10^7^/people/1 time, autologous	6 months	Child–Pugh scores, MELD scores, fatigue scale, and performance status were all improved; ascites, lower limb edema, and ALB level were improved	[115]
LT	BM-MSC	10	CI	I/II	1.5–3 × 10^6^/kg/1 time, allogeneic	12 months	No significant improvement	[116]
HBV-ACLF	UC-MSC	11	IAI	I	1 × 10^8^/people/1 time, allogeneic	24 months	Liver function levels including ALB, ALT, AST, Tbil, PT, INR, and MELD scores were all improved; the survival rate was significantly improved	[117]
ACLF	UC-MSC	24	PVI	I/II	0.5 × 10^6^/kg/3 times, autologous	72 weeks	MELD scores, Tbil, and ALT were significantly decreased; ALB, cholinesterase, prothrombin activity, and platelet counts were all increased	[118]

ACLF, acute-on-chronic liver failure; ADSC, adipose-derived MSC; ALB, albumin; ALT, alanine aminotransferase; α-SMA, α-smooth muscle actin; AST, aspartate transaminase; BM-MSC, bone marrow-derived MSC; CHE, cholinesterase; CI, central intravenous; DLC, decompensated liver cirrhosis; HBV, hepatic B virus; HCC, hepatocellular carcinoma; HCV, hepatic C virus; IAI, intrahepatic arterial injection; IL, interleukin; INR, international normalized ratio; ISI, intrasplenic injection; LC, liver cirrhosis; LF, liver failure; LT, liver transplantation; MELD, model for end-stage liver disease; PBC, primary biliary cirrhosis; PC, prothrombin concentration; PCNA, proliferating cell nuclear antigen; PI, portal injection; PVI, peripheral vein injection; PT, prothrombin time; Tbil, total bilirubin; TGF-β1, transforming growth factor-β1; TNF-α, tumor necrosis factor α; UC-MSC, umbilical cord-derived MSC

More importantly, cell engraftment efficiency, including the survival and number of MSC targeted deliveries into parenchymal liver tissues, should be considered when interpreting the therapeutic efficacy of MSC transplantation for liver diseases. Notably, by performing a series of liver biopsies after 6 months of MSC therapy in patients with cirrhosis (*n* = 25), Kantarcıoğlu et al. found that MSCs could not be delivered into liver tissues in sufficient amounts [21]. Therefore, a low cell engraftment efficiency severely affects the long-term therapeutic outcomes of MSC therapy for liver diseases. Next, we describe the cell-homing process and how to improve cell engraftment efficiency to enhance the therapeutic efficacy of MSCs for liver diseases.

## MSC transplantation and homing process in vivo

Following previous reviews, MSC homing can be divided into systemic and non-systemic [22, 23]. For non-systemic homing, MSCs were locally injected into the targeted sites. In systemic homing, MSCs are administered into the bloodstream, pass through the circulatory system, and finally, transmigrate to targeted sites. In liver diseases, MSC homing is systemic, as MSC transplantation is commonly achieved by intravenous (IV) injection via different routes, including the peripheral and hepatic portal veins. After IV transplantation, MSC are initially retained in the lungs and then, delivered to the liver, spleen, and kidney. Very few MSCs are located in other organs [24]. Although the delivery route affects travel of MSCs to the injured sites, the number of cells that could transmigrate into parenchymal liver tissues was not significantly different between portal and peripheral vein administrations [25–27]. Additionally, there were no differences in the therapeutic efficacy of MSCs between peripheral and portal vein administration in acute liver failure [25] or cirrhosis models [26]. Considering that MSC survival and homing capabilities are closely related to the therapeutic efficacy of MSC therapy, IV-injected MSC, regardless of injection site, undergo similar microenvironments in vivo and the same systemic homing process. Correlatively, it has been confirmed that systemic homing is inevitable after IV injection and involves active or passive MSC extravasation followed by chemokine-guided interstitial migration toward injured sites [23]. Similar to endogenous leukocyte migration to inflammatory sites [28, 29], systemically administered MSCs undergo rolling, activation, adhesion, crawling, and migration (Fig. 2).

As an initial step, MSC rolling is commonly facilitated by selectins expressed on endothelial cells. In 2006, Rüster et al*.* first found that the rolling behavior of MSCs bound to endothelial cells occurred in a P-selectin-dependent manner [30]. However, MSCs do not express P-selectin glycoprotein ligand 1 (PSGL-1), implying that other MSC ligands interact with P-selectin in the endothelial cells. Bailey et al*.* have identified CD24 as a candidate P-selectin ligand in adipose tissue-derived MSCs [31]. Therefore, engineering MSC surfaces with PSGL-1 and Sialyl-Lewis could increase the effectiveness of MSC therapy in multiple sclerosis [32]. Liver sinusoidal endothelial cells (LSECs) are the only gatekeepers of MSCs that homes to parenchymal liver tissue. Previously, MSC rolling was abolished by blocking CD29 (also known as VLA4, a β1-integrin) on MSCs and vascular cell adhesion molecule-1 (VCAM-1) on LSECs [33]. Hence, cell rolling during MSC therapy for liver diseases depends on CD29/VCAM-1.

Cell activation during MSC homing is usually facilitated by G protein-coupled chemokine receptors (GPCRs), which couple with cytokines secreted by wounds. Extensive evidence has shown that stromal cell-derived factor1 (SDF-1, also known as CXCL-12) in endothelial cells plays a crucial role in cell activation during MSC homing [34]. SDF-1 is also a ligand of the chemokine receptor, CXCR-4, which is commonly expressed in MSCs. Significantly, overexpression of CXCR-4 in MSCs enhanced the therapeutic effect of MSC transplantation on acute liver failure by activating the PI3K/Akt signaling pathway [35]. The number of MSC homing is closely related to SDF-1 expression in injured liver tissues [36]. Therefore, SDF-1 is an important attractant for the targeted delivery of MSCs and the SDF-1/CXCR-4 axis plays a pivotal role in MSC activation and homing. In addition to the SDF-1/CXCR-4 axis, direct interaction between other chemokines and receptors, including CCL-2/CCR-2 [37] and cannabinoid receptor-1 [38], is also involved in the cell engraftment process of MSC therapy for liver diseases. Hence, the expression of the GPCRs plays an important role in cell activation during MSC therapy for liver diseases, but the details of the underlying mechanisms require further exploration.

MSC adhesion is facilitated by integrins. Semon et al*.* showed that MSC adhesion to endothelial cells, including those in the pulmonary artery, cardiac-derived microvasculature, and umbilical veins, is markedly reduced by β5-integrin antibodies [39]. In liver diseases, Aldridge et al*.* found that blocking the β1-integrin (CD29) on MSCs significantly reduced their adhesiveness to LSECs, whereas GPCRs, including CCR-4, CCR-5, and CXCR-3, made little contribution to MSC adhesion [33]. Therefore, integrin expression in MSCs affects their adhesion capability during MSC homing.

MSCs crawl on the surface of endothelial cells along with the establishment of firm endothelial adhesion. Cell crawling, the movement along extracellular substrates or matrices (e.g., inner vessel walls), requires exogenous factors, including fluid force, and chemokines at targeted sites [23, 40]. Chamberlain et al*.* found that shear stress and CXCL-9 significantly enhanced MSC crawling capability on endothelial cells in vitro [29]. Lateral cell crawling is accompanied by MSC polarization, which is initiated by the crosstalk between FROUNT and CCR-2, followed by CCR-2 clustering, leading to cytoskeletal reorganization and further endothelial migration [41].

To accomplish endothelial migration, MSCs must penetrate the barriers of the endothelial cell layers by secreting MMPs (including MMP-1, MMP-2, MMP-9, and MT1-MMP), which can degrade the basement membrane of endothelial cells [42, 43]. The MMP activity is commonly regulated by TIMP-1 [44], microRNAs [45], and inflammatory factors (e.g., IL-1β [46], TGF-β1 [47], and TNFα [43]). Apart from MMPs, other cytokines including CXCR-3 and urokinase-type plasminogen activator induced by inflammatory factors such as IL-1β and IL-17, are also involved in the trans-endothelial migration of MSCs [48, 49].

### Strategies for enhancing MSC survival and homing capability in liver diseases

**Fig. 3:**
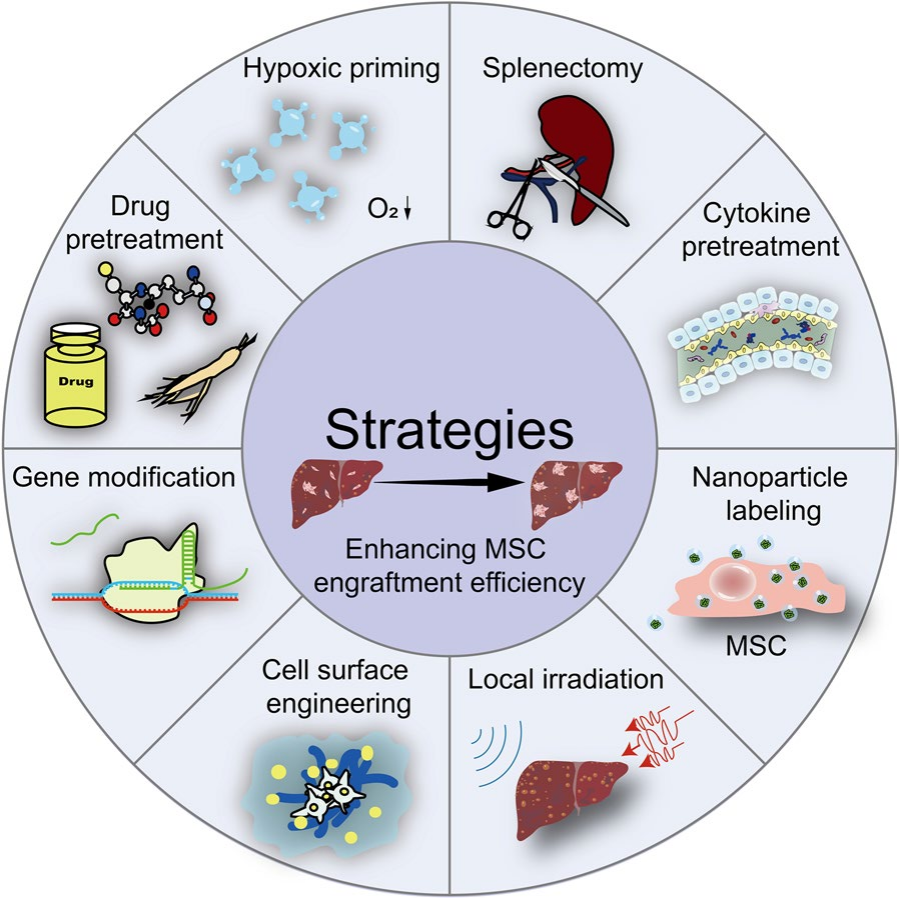
Strategies for enhancing MSC survival and homing capability. The current strategies including hypoxic priming, drug pretreatment, gene modification, cell surface engineering, cytokine pretreatment, splenectomy, nanoparticle labeling, and local irradiation have been used to improve MSC survival and cell-homing capability. Figure designed by Adobe Illustrator CC 2018

After administration into the bloodstream, MSC will encounter a range of conditions that can influence their survival. The optimal constant conditions that support growth in vitro give way to *more* inclement, complex conditions in vivo, including low oxygen tensions, fluid pressure stress, and interaction with whole blood components. As a result, many MSCs die in the blood circulation after IV transplantation. Furthermore, following the tortuous homing process, the existing surviving MSCs continue to be subjected to challenging conditions such as hypoxia, oxidative stress, and inflammation in the targeted sites, leading to continuous cell death, such that only a small number of viable MSCs populate the parenchymal liver tissues. The cell attrition dramatically reduces theoretical functions of MSC transplantation in liver diseases. Considering that cell survival and cell-homing capability are closely related to MSC engraftment efficacy, it is necessary to further improve MSC survival and homing capabilities and to maximize the therapeutic efficiency of MSC therapy in liver diseases. Next, we summarized the current strategies for enhancing cell survival and homing capability of MSC transplantation (Fig. 3).

### Strategies for enhancing MSC survival in vivo

#### Hypoxic priming

Generally, human arterial blood contains 12.3% O_2_, venous blood contains 5.3% O_2_, and the liver tissue contains approximately 4.04% O_2_ (30.7 mmHg of O_2_) [50]. Comparing in vitro expansion of normoxic cultured MSCs (NC-MSCs, approximately 19.95% O_2_), the oxygen dissolution in vivo and the oxygen content in liver tissues and the circulatory system are extremely low. After short-term hypoxic exposure, NC-MSCs were prone to death due to upregulation of Sug1, and the inactivation of 26S proteasome, leading to increased immunogenicity [51, 52], and inducing cell apoptosis [53]. Additionally, NC-MSC stemness is easily lost during extensive amplification in vitro [54]. Hence, it is difficult to adapt in vivo expansion of NC-MSCs to dramatic changes in oxygen pressure. To improve the ability of MSCs to adapt to changes in oxygen, hypoxic priming, an in vitro preconditioning method, has been used to increase their survival in vivo [55, 56]. Hypoxic priming can increase autocrine or paracrine factor secretion by MSCs, including IL-6, TNFα, HGF, VEGF, and prostaglandin E synthase, which promotes liver regeneration and reduces hepatocyte apoptosis [57]. In addition, it prevents MSC senescence by promoting autophagy [58], and downregulating p16, p53, and p21 [59, 60]. Therefore, hypoxic priming has been used to enhance the outcomes of MSC therapy for liver diseases [61] (Table 2).

**Table 2:** Hypoxic preconditioning improves therapeutic outcomes of MSC transplantation for liver diseases

Oxygen pressure	Model	Derivation	Therapeutic effect	References
1% O_2_	Rat, 85% hepatectomy	Rat bone marrow	Increased survival and liver regeneration	[53]
1% O_2_	Mouse, 70% hepatectomy	Human adipose tissue	Accelerated liver regeneration and liver function recovery	[56]
1% O_2_	Mouse, liver fibrosis	Human bone marrow	Accelerated liver function recovery	[55]
1% O_2_	Mouse, hepatectomy	Human adipose tissue	Reduced hepatocyte apoptosis	[61]
5% O_2_	Mouse, liver cirrhosis	Human bone marrow	Enhanced liver regeneration and reduced hepatocyte apoptosis	[57]
2–3% O_2_	Mouse, liver ischemia	Human bone marrow	Enhanced liver regeneration	[119]

AKT, protein kinase B; CXCR-4, C-X-C motif chemokine receptor 4; HGF, hepatocyte growth factor; IL-6, interleukin-6; MSC, mesenchymal stem cell; PGE-2, prostaglandin E2; TNF-α, tumor necrosis factor α; VEGF, vascular endothelial growth factor

#### Drug pretreatment

Accumulating evidence suggests that oxidative stress characterized by the excess generation of reactive oxygen species is a key factor in the low cell survival rate of transplanted MSCs [62]. Antioxidant drugs have been used to overcome oxidative stress and enhance MSC survival in vitro (Table 3). Indeed, our group found that a low dose of reduced glutathione (GSH) and melatonin could be used to preserve MSC functions (including cell proliferation, and stemness) and to reduce cell senescence during long-term in vitro passaging [63]. Importantly, antioxidant pretreatment increased MSC survival by reducing cell apoptosis in an H_2_O_2_ injury model [64] and enhanced therapeutic outcomes of MSC therapy for liver fibrosis [64, 65]. Pretreatment with other antioxidants, including edaravone [66], zeaxanthin dipalmitate [67], and vitamin E [68], can also be used to enhance MSC survival and therapeutic efficacy for liver failure.

**Table 3:** Antioxidant drug pretreatment improves MSC survival

Drug	Concentration	Derivation	Mechanism	References
GSH	10 μM	Mouse adipose tissue	Reduced H_2_O_2_-induced injuries by reducing ROS generation and cell apoptosis	[56, 63]
Melatonin	10 μM	Mouse adipose tissue	Reduced H_2_O_2_-induced injuries by reducing ROS generation and cell apoptosis	[56, 63]
Edaravone	20 μM	Human umbilical cord	Reduced LPS/H_2_O_2_-induced injuries by improving cell viability and by reducing cell apoptosis	[56]
Zeaxanthin dipalmitate	0.5 μM	Human adipose tissue	Reduced LPS/H_2_O_2_-induced injuries by reducing ROS generation, cell apoptosis and inflammation	[67]
Ginsenoside Rg1	50 μM	Mouse bone marrow	Reduced cell senescence and improved antioxidant capacity of MSCs via NRF-2 and PI3K/Akt signaling	[120]
Wedelolactone	3.18–318 μM	Rat bone marrow	Reduced •OH- or O_2_^−^-induced injuries by improving cell viability	[121]
Astaxanthin	16 μM	Human adipose tissue	Reduced H_2_O_2_-induced injuries by reducing ROS generation and cell apoptosis	[122]
Exendin-4	20 nM	Rat adipose tissue	Reduced H_2_O_2_-induced cell apoptosis via PI3K/Akt–Sfrp2 pathways	[123]
Taxifolin	1–100 μg/mL	Rat bone marrow	Reduced •OH -induced injuries by improving cell viability	[124]
Chlorogenic acid	100 μM	Rat bone marrow	Reduced H_2_O_2_-induced cell apoptosis via PI3K/AKT signal and FOXO family genes	[125]
Vitamin E	100 μM	Human umbilical cord	Enhanced MSC survival by reducing oxidative stress	[68]

FOXO, forkhead box O; GSH, glutathione; LPS, lipopolysaccharides; MSC, mesenchymal stem cell; NRF-2, nuclear factor erythroid 2-related factor 2

Similar to oxidative stress, inflammation is another factor affecting MSC survival in vivo. We previously used a ratiometric near infrared-II fluorescence probe to track MSC viability and found that dexamethasone pretreatment could improve MSC cell survival and enhance the hepatic protection of MSC transplantation for liver fibrosis [12]. Moreover, juzentaihoto, a chemical drug with both anti-inflammatory and anti-oxidative functions, has also been used to improve cell survival and to enhance the therapeutic efficiency of MSC transplantation for liver cirrhosis [69]. Considering that some antioxidant and anti-inflammatory drugs (e.g., GSH) have been used clinically in patients with ESLD, drug pretreatment is a promising clinical strategy for enhancing MSC survival and therapeutic efficacy for liver diseases.

#### Gene modification

Given that miR-210 is closely involved in cell survival under hypoxia or oxidative stress, its overexpression has been used to enhance MSC survival under hypoxic conditions [67] or oxidative stress induced by H_2_O_2_ [70], thereby improving the repair function of MSC transplantation. Overexpression of anti-apoptotic, antioxidant, or pro-survival genes including BCL-2 [71, 72], Akt1 [73], HGF [74], GATA-4 [75], and erythropoietin (EPO) [76], significantly enhanced MSC survival in vitro and in vivo*.* In addition, down-regulation of miR-34a [77], and miR-16 [78] enhanced MSC survival by reducing apoptosis. Therefore, modifying gene expression to reduce cell apoptosis and/or improve the adaptability to hypoxia and oxidative stress is an alternative method for enhancing MSC survival in vivo.

### Strategies for enhancing MSC homing capability in vivo

#### MSC modification in vitro

##### Gene modification

The entire process of MSC homing is medicated by the crosstalk between ligands and receptors. Increasing the expression ligands or receptors on MSCs improves their homing capability. Overexpression migration-related genes, including CXCR-4 [79], CCR-2 [80], CXCL-9 [81], and c-Met [82], have been used to increase MSC homing. Gene modification also significantly enhances the therapeutic efficacy of MSCs for acute or chronic liver diseases (Table 4).

**Table 4:** Gene modification for enhancing cell-homing capability of MSC transplantation in liver diseases

Cell type	Gene	Vector	Treatment	Model	Mechanism	References
BM-MSC	CXCR4	Adenovirus	Overexpression	LT	By CXCR-4/SDF-1α pathway and by inhibiting liver enzyme release and caspase-3 expression	[79]
BM-MSC	Androgen receptor	RNAi	Knockout	LC	By regulating IL-1R/IL-1Ra signaling	[126]
BM-MSC	EPO	Lentivirus	Overexpression	Liver fibrosis	By suppressing TGF-β1 and IL-6 expression and upregulating MMP-9 expression	[76]
AF-MSC	IL-1Ra	Lentivirus	Overexpression	Liver failure	By inhibiting hepatic inflammatory and reducing hepatocyte apoptosis	[127]
PD-MSC	PRL-1	4D AMAXA Nucleofector™ system	Overexpression	LT	By regulating integrin signal pathway and decreasing hsa-miR-30a-5p expression	[128]
UC-MSC	CXCL9	Lentivirus	Overexpression	Liver fibrosis	By enhancing MSC adhesion, crawling and spreading	[81]
BM-MSC	C-met	Lentivirus	Overexpression	ALF	By enhancing HGF/c-Met pathway to improve MSC homing	[82]
UC-MSC	CCR2	Lentivirus	Overexpression	ALF	By enhancing MSC homing through regulating CCR-2/CCL-2 axis	[37]
BM-MSC	FGF4	Lentivirus	Overexpression	LC	By improving MSC proliferation and migration	[129]
BM-MSC	HGF	Adenovirus	Overexpression	LC	By improving MSC homing	[130]
BM-MSC	hFoxa2	Lipofectamine	Overexpression	LC	By promoting the incorporation of MSCs into liver grafts	[131]
AMM	IL-10	Neon transfection system	Overexpression	LC	By enhancing MSC retention	[132]
ADSC	BCAT1	Adenovirus	Overexpression	ALF	By enhancing MSC retention	[133]
UC-MSC	VEGF_165_	Adenovirus	Overexpression	ALF	By improving MSC homing	[134]

ADSC, adipose -derived MSC; AF-MSC, amniotic-fluid–derived MSC; ALF, acute liver failure; AMM, amniotic mesenchymal stem cell; BCAT1, branched‐chain amino acid transaminase‐1; BM-MSC, bone marrow-derived MSC; CCR-2, CC chemokine receptor 2; C-met, cellular-mesenchymal epithelial transition factor; CXCL-2, C-X-C chemokine ligand 2; CXCL9, C-X-C chemokine ligand 9; CXCR-4, C-X-C chemokine receptor type 4; EPO, erythropoietin; FGF4, fibroblast growth factor 4; hFoxa2, human Forkhead box A2; HGF, hepatocyte growth factor; HSC, hepatic stellate cell; IL-1Ra, interleukin-1-receptor antagonist; IL-6, interleukin-6; IL-10, Interleukin 10; LC, liver cirrhosis; LT, liver transplantation; MMP-9, matrix metalloproteinase 9; PD-MSC, placenta-derived MSC; PRL-1, phosphatase of regenerating liver-1; TGF-β1, transforming growth factorβ1; UC-MSC, umbilical cord blood-derived MSC; VEGF_165_, vascular endothelial growth factor 165

#### Cell surface engineering

Cell surface engineering to decorate a targeted molecule on the cell surface has been used to enhance MSC delivery to the target sites [83]. Previously, human adipose tissue-derived MSC surfaces were engineered with lipid-conjugated heparin to increase hepatic homing of MSCs and improve MSC therapy for acute liver failure [84–86]. Given that LSECs are a specific permeable barrier of the hepatic sinusoidal endothelium for trans-endothelial migration of MSC transplantation, we used bioorthogonal click chemistry to modify the MSC surface with an LSEC-targeted peptide (RLTRKRGLK) to increase MSC homing capability to enhance MSC therapy for acute liver failure and liver fibrosis [87]. Importantly, neither heparin-functionalization nor the bioorthogonal click chemistry approach affected the biological characteristics of the MSCs. Therefore, these cell surface engineering strategies are a promising for enhancing MSC homing capability.

### Cytokine pretreatment

Pretreatment with cytokines, including IL-17 [88] and HGF [89], improved MSC migration and homing ability in vivo. Recently, Nie et al*.* found that IL-1β pretreatment increased CXCR-4 expression and enhanced MSC homing capability and therapeutic outcomes for acute liver failure [46]. Pretreatment with TGFβ1 enhanced the homing and engraftment of MSCs to human and murine hepatic sinusoidal endothelia in vivo and in vitro, which was mediated by increased expression of CXCR-3. In particular, pretreatment with cytokine can enhance the anti-inflammatory effects of MSC therapy in acute liver injury [90]. Because cytokines can be easily controlled within the GMP grade, cytokine pretreatment provides translational potential for improving the MSC homing capacity for liver diseases.

### Nanoparticle labeling

Nanoparticle-based imaging has been widely used for in vivo assessment of MSC biodistribution. Huang et al*.* developed an iron-based nanocluster for MSC labeling and found that it enhanced MSC migration by promoting CXCR-4 expression [91]. Similarly, Vitale et al*.* developed silica nanoparticles (SiO_2_-NPs) for MSC tracking and found that their internalization enhanced MSC migration by increasing CXCR-4 expression [92]. Hence, silica nanoparticle labeling is a novel method for improving the homing capabilities of MSCs. Nevertheless, the detailed mechanism and safety profile of nanoparticle labeling for increasing CXCR-4 expression remain unknown.

### Host environment regulation

#### Splenectomy

Portal hypertension is a typical physical condition aggravated by cirrhosis. Previously, it was suggested that the flow shear stress benefits the osteogenic, cardiovascular, chondrogenic, adipogenic, and neurogenic differentiation of MSCs [93]. However, high shear stress and portal hypertension hamper the adhesion and migration of MSCs. Splenectomy is a therapeutic option for increasing platelet count and promoting liver regeneration in patients with portal hypertension and cirrhosis. In particular, Tang et al*.* found that splenectomy enhanced MSC homing capability and therapeutic efficacy for cirrhosis of the liver in rats by upregulating of SDF-1 and HGF [94]. Nevertheless, the detailed mechanism and safety of this approach still need to be verified before clinical application.

#### Transient local irradiation

Considering that transient local irradiation (TLR) can disturb the LSEC barrier and inhibit the phagocytic function of Kupffer cells, TLR has been used to enhance hepatocyte engraftment in hepatectomized mice [95]. Inspired by this, Shao et al*.* used hepatic TLR to enhance MSC homing and therapeutic outcomes for thioacetamide-induced fibrosis in rats [96]. Hence, TLR is an alternative method for improving the MSC homing capability. However, this approach increases the risk of tissue injury, and its clinical benefits should be fully evaluated before further application.

## Conclusions and future directions

Here, we review the whole cell-homing process of MSC transplantation and the current clinical status of MSC therapy for liver diseases, emphasizing that low cell engraftment efficiency is a major challenge to the use and the long-term therapeutic efficacy of MSC therapy. We also highlighted that cell survival and MSC transmigration into the parenchymal liver tissues is closely related to the efficiency of MSC engraftment. Therefore, we summarized the current strategies to enhance cell survival and homing capability for MSC transplantation in liver diseases. Nevertheless, there are many unanswered questions regarding the safety and the clinical potential of these strategies. First, although pretreatment with drugs, hypoxia, and cytokines can improve MSC survival or homing capability, they also affect the paracrine functions of MSCs; hence, future studies are still needed. Second, modifying MSCs to enhance their homing capability through gene editing, nanoparticle labeling, or chemical methods is an alternative approach for enhancing MSC engraftment efficiency; however, biosafety issues and how to achieve GMP-grade cell production requires further exploration. Third, splenectomy or TLR poses an external risk to patients, and the clinical benefit should be fully verified before implementation in clinical settings. Finally, the current strategies are supported by in vitro and animal studies, but their clinical translational potentials for improving cell survival and homing capability of MSC therapy in liver diseases remain to be tested.

Apart from cell survival and homing capability, there are a large number of variables, including the heterogeneity of MSCs derived from different tissues and individual differences in patients, which affect cell engraftment efficiency and personalized MSC therapy for liver diseases. There is a clear need to develop personalized models to address therapeutic efficacy of MSC transplantation in liver diseases.

## Data Availability

Not applicable.

## Abbreviations

α-SMA: α-Smooth muscle actin
ACLF: Acute-on-chronic liver failure
ADSC: Adipose-derived MSC
AF-MSC: Amniotic-fluid-derived MSC
ALB: Albumin
ALF: Acute liver failure
ALT: Alanine aminotransferase
AKT: Protein kinase B
AMM: Amniotic mesenchymal stem cell
AST: Aspartate transaminase
BCAT1: Branched‐chain amino acid transaminase‐1
BM-MSC: Bone marrow-derived MSC
CCR-2: CC Chemokine receptor 2
CD29: β1-Integrin
CHE: Cholinesterase
CI: Central intravenous
C-met: Cellular-mesenchymal epithelial transition factor
CXCR-4: C-X-C chemokine receptor type 4
CXCL-2: C-X-C chemokine ligand 2
CXCL-9: C-X-C chemokine ligand 9
DLC: Decompensated liver cirrhosis
ESLD: End-stage liver diseases
EPO: Erythropoietin
FGF4: Fibroblast growth factor 4
FOXO: Forkhead box O
GSH: Glutathione
HBV: Hepatic B virus
HCC: Hepatocellular carcinoma
HCV: Hepatic C virus
hFoxa2: Human Forkhead box A2
HGF: Hepatocyte growth factor
HSC: Hepatic stellate cell
IAI: Intrahepatic arterial injection
IL-1Ra: Interleukin-1-receptor antagonist
IL-6: Interleukin-6
IL-10: Interleukin 10
INR: International normalized ratio
ISI: Intrasplenic injection
IV: Intravenous injection
LC: Liver cirrhosis
LF: Liver failure
LPS: Lipopolysaccharides
LT: Liver transplantation
LSECs: Liver sinusoidal endothelial cells
MELD: Model for end-stage liver disease
MMP-9: Matrix metalloproteinase 9
MSC: Mesenchymal stem cell
NRF-2: Nuclear factor erythroid 2-related factor 2
OS: Overall survival
PBC: Primary biliary cirrhosis
PC: Prothrombin concentration
PCNA: Proliferating cell nuclear antigen
PGE-2: Prostaglandin E2
PD-MSC: Placenta-derived MSC
PI: Portal injection
PRL-1: Phosphatase of regenerating liver-1
PSGL-1: P-selectin glycoprotein ligand 1
PT: Prothrombin time
PVI: Peripheral vein injection
ROS: Reactive oxygen species
SDF-1: Stromal cell-derived factor1
Tbil: Total bilirubin
TGFβ1: Transforming growth factorβ1
TLR: Transient local irradiation
TNF-α: Tumor necrosis factor α
UC-MSC: Umbilical cord-derived mesenchymal stem cell
VCAM-1: Vascular cell adhesion molecule-1
VEGF: Vascular endothelial growth factor
VEGF_165_: Vascular endothelial growth factor 165
